# Research techniques to expand the diagnosis, macroelimination, and microelimination of hepatitis C in local contexts

**DOI:** 10.1590/0037-8682-0150-2024

**Published:** 2024-11-15

**Authors:** Geisa Perez Medina Gomide, Fernanda Carolina Camargo, Cristina da Cunha Hueb Barata de Oliveira

**Affiliations:** 1 Universidade Federal do Triângulo Mineiro, Programa de Pós-Graduação Stricto Sensu em Medicina Tropical e Infectologia, Uberaba, MG, Brasil.; 2 Universidade Federal do Triângulo Mineiro, Gerência de Ensino e Pesquisa do Hospital de Clínicas filial Ebserh, Uberaba, MG, Brasil.; 3 Universidade Federal do Triângulo Mineiro, Departamento de Clínica Médica, Uberaba, MG, Brasil.

**Keywords:** Hepatitis C, Epidemiologic studies, Investigative techniques, Sociocultural territory, Risk groups, Social determination of health

## Abstract

**Background:**

Local studies are essential to determine the prevalence and risk factors of hepatitis C.

**Methods:**

We conducted a mixed methods study aimed at strengthening hepatitis C elimination efforts in Brazil, with concomitant triangulation of techniques and integration of qualitative and quantitative approaches.

**Results:**

The study resulted in the development of the following technical-technological products: a) institutional documents, b) organization of committees, c) mobilization strategies, d) models for improving intersectoral awareness, e) decision-making flowcharts, f) forms, and g) health service protocols and search strategies.

**Conclusions:**

The application of these research techniques generated valuable knowledge that could be adopted in various regions across Brazil, particularly in areas with economic and sociocultural diversity.

Viral hepatitis is the leading cause of chronic infections and mortality, affecting millions of individuals worldwide. Patients in the acute phase of the disease are often asymptomatic; however, hepatitis C virus (HCV) and hepatitis B virus (HBV) accounted for 96% of deaths associated with viral hepatitis in 2015. These viruses persist as significant contributors to global morbidity and mortality^1^.

The World Health Organization (WHO) has developed coordinated actions to eliminate these diseases. The strategies were aimed at reducing the incidence and mortality by 90% and 65%, respectively, with 2015 serving as the baseline year. A universal screening test for HCV infection is recommended for all adults. Macroelimination involves immediate treatment with direct-acting antivirals, whereas, microelimination involves continuous screening based on the identified risk behaviors and exposures, along with the prompt treatment of individuals who tested positive for HCV infection^2^.

In recent years, significant global progress has been made in the elimination of hepatitis. Owing to the tenfold increase in HCV treatment rates compared with the baseline strategy, HCV mortality has declined since 2019. However, approximately 78.6% of HCV infections remain undiagnosed^3^.

HCV infection is a notable cause of morbidity in low- and middle-income countries. In Latin America, the prevalence rates were 0.5% in the central and Andean regions, 0.6% in the southern region, and 0.9% in the tropical regions. The use of injectable drugs and blood transfusions without adequate safety standards along with high-risk sexual behaviors are the primary modes of transmission. A recent meta-analysis of 23 studies with 11,419 patients revealed an HCV infection prevalence of 57% among individuals with injection drug use in Latin America^4^.

Despite being one of the largest economies worldwide, Brazil experiences significant income inequality, leading to disparities in access to public health and education. Although the government, through the Unified Health System, covers all direct medical costs associated with HCV infection, many Brazilians are unable to afford other significant direct and indirect nonmedical expenses related to screening and the costs associated with accessing and adhering to healthcare services from the initial follow-up to recovery^5^.

In certain regions, HCV infection is more prevalent among those who inject drugs, those who are stigmatized, and are reluctant to undergo testing for infection. Furthermore, the difficulty in accessing tests and treatments for at-risk populations continues to pose a problem. The proposed solutions to these barriers include decentralized and integrated care and increased access to generic medicines, which must be implemented globally to achieve effective results^1^.

In August 2023, nine ministries in Brazil collaborated to develop strategies aimed at eliminating diseases that primarily affected socially vulnerable populations. The Interministerial Committee for the Elimination of Tuberculosis and Other Socially Determined Diseases (CIEDDS), coordinated by the Ministry of Health, aims to develop coordinated actions to promote social inclusion and provide comprehensive care for people with such diseases. The work plan addresses 11 diseases, including viral hepatitis, in alignment with the United Nations Sustainable Development Goals. The committee asserts that the CIEDDS will serve as an example for other countries and highlights the significant influence of social determinants of health on the transmission and persistence of various infectious diseases. Interministerial coordination is deemed essential, as the solution to these diseases extends beyond the health sector alone. It is based on the premise that ensuring access to health care alone is insufficient to achieve the WHO goals. Therefore, intersectional public policies aimed at addressing health-related and social inequalities should be proposed^6^.

To support these efforts, reflecting on the role of research techniques and study designs in enhancing public health programs is essential for addressing diseases in local contexts. Therefore, investing in local research remains crucial for identifying the risk factors and understanding the prevalence of these conditions. Thus, this study aimed to explore research techniques that could expand the diagnosis and macro- and microelimination of HCV infections in local contexts, focusing on the Southern Triangle macro-region of Minas Gerais in 2024. This presentation outlines the strategies employed in the primary studies.

Scientific literature defined the prevalence of the virus at the international and national levels. However, only a few studies have described its prevalence in specific regions or locations. This knowledge is essential for guiding actions, such as case tracking, improving access to diagnosis and treatment in primary care, and developing strategies for priority populations that account for the largest number of cases. In this context, promoting research strategies that foster intersectoral integration is essential.

A growing trend emphasizes the reconciliation of coordinated research methods. Mixed methods offer a comprehensive approach, combining predetermined research techniques to gather multiple types of data through statistical and textual analyses. This approach allows for the expansion of research data, incorporating open records from observations and surveys, followed by more in-depth exploratory interviews. Our investigation employed a mixed methods approach based on the assumption that the collection of different types of data facilitates a more comprehensive understanding of the research problem^7^.

Three key characteristics of mixed methods were highlighted: methodological eclecticism, paradigmatic pluralism, and a focus on addressing a specific research question when determining the appropriate methods to be used in any study. Importantly, methodological combinations should directly respond to the challenges encountered in the investigative process^7^. From the outset of the investigation, the researcher’s reflections and actions shaped various aspects of the methodological approach, which, according to the mixed methods perspective, were illustrated in a flowchart of the study design (Figure 1). 

**FIGURE 1:**
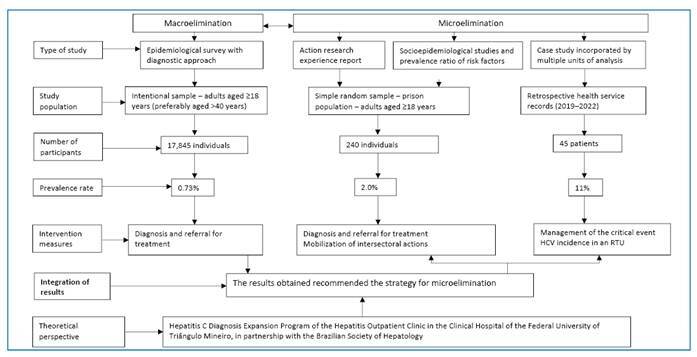
Flowchart of the mixed methods study design with the corresponding results. Uberaba, Minas Gerais, 2023. Source: prepared by the author, 2023. **HCV:** hepatitis C virus; **RTU:** renal therapy unit.

Focusing on macroelimination, a survey was conducted in 17,845 individuals from the local community to identify the primary risk groups. Among the participants, 0.73% tested positive for anti-HCV, of whom 79.3% were born between the 1950s and 1970s and 24% had moderate to advanced liver fibrosis or cirrhosis. The most frequently reported forms of transmission included drug use, having multiple sexual partners and receiving transfusions, hemodialysis, or organ transplantation^8^ ^,^ ^9^ (Figure 1).

Subsequently, action research was conducted in the local penitentiary as a strategy for the microelimination of HCV infection. Epidemiological inquiries and anti-HCV testing were conducted on a simple random sample of 240 prisoners, revealing a prevalence rate of 2%. Prevalence Ratio (PR) and 95% Confidence Interval (CI95%) were used. The use of injectable drugs (PR: 14.75, PR CI95%: 2.09-104.28), being born between 1951 and 1980 (PR: 9.28, PR CI95 %: 1.06-81.57), and co-infection with the HBV (PR: 10.75, PR CI95%: 1.66-69.65) were identified as significant factors associated with the prevalence of the virus. Furthermore, 76.6% of the participants were men, with an average age of 33.8 years and 80.5% possessing incomplete secondary education. Additionally, 93.8% were heterosexual, had multiple partners, and exhibited inadequate condom use. Approximately 70% of the participants reported using non-injectable drugs, 72.9% reported high alcohol consumption, 70.4% were repeat offenders, and 75.8% had body tattoos^10^ ^-^ ^12^ (Figure 1).

A case study was conducted in the hemodialysis unit of a local hospital, following a reported increase in the incidence of HCV infection in 2019. Of the 45 patients monitored, 6 tested positive for anti-HCV antibodies. These patients had been exposed to contaminated medical equipment, contaminated objects, or the inadequately sanitized hands of medical personnel. Preventive measures were adopted, and routine techniques were revised. The Situational Analysis Committee guided event management. All patients received treatment, and no new cases were detected^13^ (Figure 1). 

Based on a mixed method study design, the proposed research techniques were aimed at strengthening the macro- and microelimination of HCV in local contexts through the triangulation of methods and the integration of qualitative and quantitative approaches in a study conducted in Brazil. This approach allows the integration of both qualitative and quantitative approaches, by incorporating community-based epidemiological surveys^8^ ^,^ ^9^, action research targeting populations with the highest prevalence and vulnerability in the local context^10^ ^,^ ^11^, and reports on research management experiences in vulnerable and critical scenarios^12^ ^,^ ^13^.

The research techniques employed in this mixed methods study aimed to advance several key objectives: *a)* continuous training of the primary care network regarding priority risk groups and treatment facilities; *b)* the development of intersectoral health projects, focusing on public health institutions and the prison system; *c)* the establishment of testing policies for individuals admitted to prisons and the immediate treatment of those diagnosed; *d)* the annual testing of individuals deprived of liberty in risk situations (such as drug users); *e)* the provision of continued care to prisoners reintegrating into the community; *f)* raising awareness and motivating nephrologists to provide HCV care in dialysis facilities; and *g)* carrying out tests upon patient admission to the hemodialysis units and ensuring immediate treatment of individuals with positive results^10^ ^-^ ^13^.

This proposal on research techniques was developed based on research conducted by the HCV Diagnosis Expansion Program group at the Hepatitis Outpatient Clinic of the Clinical Hospital of the Federal University of Triângulo Mineiro. The focus lies on diagnosing HCV carriers in Uberaba, understanding the epidemiology, identifying the main risk groups, and defining the strategies for the macro and microelimination of the HCV.

The methodological approach outlined here led to the design of this research using mixed methods and resulted in the creation of various technical-technological products integral to its development: *a)* institutional documents, *b)* organization of committees, *c)* mobilization strategies, *d)* models for improving intersectoral awareness, *e)* decision-making flowcharts, *f)* forms, *g)* health and research service protocols. These products can be shared nationally and internationally to facilitate the expansion of HCV diagnosis and elimination efforts by 2030.

Conducting the survey in a specific territory to reach a larger population facilitated the identification of priority groups for new coping and microelimination research in the region. Therefore, implementing similar strategies are appropriate for managing problems in different territories.

A notable technical contribution of this research design is its ability to guide testing within the general population, enabling the prioritization and ranking of the most prevalent risk groups based on geographic location. Given Brazil’s economic and sociocultural diversity, in addition to the widespread health inequalities, this strategy should be implemented in various regions of the country.

This methodological approach also aims to optimize health resources within a Universal System while promoting equity by prioritizing care, coping, and microelimination groups according to the context of each location. Another crucial aspect of this study is mobilization. In this study, intersectoral coordination was essential for addressing the needs of special populations. The research would not have been possible without the involvement of the State Secretariat of Justice and Public Security of Minas Gerais, the board of the Penitentiary Professor Aluízio Ignácio de Oliveira, the Municipal Health Department of Uberaba/Minas Gerais, the Clinical Hospital of the Federal University of Triângulo Mineiro - EBSERH branch, the Brazilian Society of Hepatology, and all Rotary Clubs in Uberaba/Minas Gerais. Addressing this issue, as outlined here, extends beyond the confines of the clinical office.

Coordinated action among different sectors is essential, making partnerships crucial for success. This study described mobilization strategies that represent a significant and unique contribution to guiding the adoption of these measures by approaching vulnerable groups in other national territories.

Despite the broad scope of these challenges, they underscore the need to implement policies that facilitate intersectoral dialogue to support the most vulnerable populations. By integrating efforts across all stages, this study developed an instructional design/model to guide the expansion of HCV diagnosis. The approach is organized into distinct stages: conducting a survey to identify the overall prevalence and the most vulnerable groups in specific localities, and implementing intersectoral mobilization strategies to enable microelimination within these groups.

Notably, this study developed a comprehensive database that can support future research aimed at analyzing individuals’ living conditions, health status, and the correlates of HCV infection. The database comprised demographic variables, sexual experiences, family history, alcohol and drug use, and other risk factors for exposure to hepatitis viruses. For specific groups, the database captures specific variables, such as the duration of dialysis treatment and frequency and length of incarcerations. The questionnaires used in the surveys can also serve as valuable tools for screening populations with risk factors and vulnerable conditions. Institutional protocols developed from these findings can be utilized to guide management and elimination strategies.

The research model for expanding diagnosis and achieving macro- and microelimination in vulnerable populations generated valuable insights that guided its recommendations.

## ETHICAL CONSIDERATIONS

In accordance with the Declaration of Helsinki and Brazilian ethical standards, the studies were approved by the Research Ethics Committee of the Universidade Federal do Triângulo Mineiro Hospital, in accordance with Resolution no. 466/2012, which deals with human research (approval No. 3.930.299; No. 3.918.981; No. 2.394.876).

## References

[1] Black AP, Wallace J, Binka M, Butt ZA (2023). The challenges of viral hepatitis elimination: a global response to a global problem. BMC Public Health.

[2] Villela-Nogueira CA, Ferraz MLG, Pessoa MG, Souto FJD, Nabuco LC, Coelho HSM (2023). Choosing wisely recommendations regarding the top five list of procedures to avoid in the treatment of viral hepatitis: A position statement from the Brazilian Society of Hepatology endorsed by the Latin American Association for the Study of the liver. Ann Hepatol.

[3] Cui F, Blach S, Mingiedi CM, Gonzalez MA, Alaama AS, Mozalevskis A (2023). Global reporting of progress towards elimination of hepatitis B and hepatitis C. Lancet Gastroenterol & Hepatol.

[4] Magri MC, Manchiero C, Dantas BP (2023). Hepatitis C among people who inject drugs (PWID) in Latin America and the Caribbean: a meta-analysis of prevalence over three decades. J Stud Alcohol Drugs.

[5] Bittencourt PL, Iasi MSF, Viana MV, Crespo DM, Emerim E, Borges PSA (2022). Poor linkage to care may compromise the Brazilian plan for hepatitis C elimination. J Viral Hepat.

[6] Maciel ELN, Sanchez MN, Cruz AM, Cravo DB, Lima NVT (2024). Brazil’s Pivotal Moment in Public Health: Establishing the Interministerial Committee (CIEDDS) for the Elimination of Tuberculosis and Socially Determined Diseases. Rev Soc Bras Med Trop.

[7] Creswell JW (2007). Projeto de pesquisa métodos qualitativo, quantitativo e misto.

[8] Gomide GPM, Melo CB, Santos VS, Salge VD, Camargo FC, Pereira GA (2019). Epidemiological survey of hepatitis C in a region considered to have high prevalence: the state of Minas Gerais, Brazil. Rev Soc Bras Med Trop.

[9] Gomide GPM, Molina RJ, Pereira GA, Oliveira CCHB (2021). Diagnóstico precoce da hepatite C pela atenção primária à saúde. Rev Fam Ciclos Vida Saúde Contexto Soc.

[10] Gomide GPM, Teixeira MS, Pereira GA, Camargo FC, Pastori BG, Dias FF (2024). Epidemiological study of hepatitis C in people deprived of liberty. ABCS Health Sci.

[11] Gomide G, Teixeira M, Pereira G, Camargo F, Pastori B, Dias F (2023). Hepatitis C survey among the prison community in the Triângulo Mineiro region, Minas Gerais: revealing an invisible population. Concilium.

[12] Gomide GPM, Teixeira MS, Pereira GA, Camargo FC, Pastori BG, Dias FF (2022). Experiência no gerenciamento de pesquisa-ação sobre inquérito de hepatite C junto à comunidade carcerária. Cien Saude Colet.

[13] Gomide GPM, Pereira LHM, Camargo FC, Rodrigues LM, Souza RS, Melo IV (2023). Measures to contain the transmission of hepatitis C in a chronic kidney care hospital unit in the Triângulo Mineiro in Brazil: A case study. Int J Public Health.

